# Intraventricular Glioblastoma in a Neonate: A Case Report and Literature Review

**DOI:** 10.7759/cureus.73797

**Published:** 2024-11-16

**Authors:** Tala AlSindi, Khalid T Alghamdi, Abdulaziz M Alghamdi, Alaa Samkari, Hussam Kutub

**Affiliations:** 1 Department of Neurosurgery, King Abdulaziz University Hospital, Jeddah, SAU; 2 Department of Neurosurgery, King Faisal Specialist Hospital and Reseach Centre, Riyadh, SAU; 3 College of Medicine, King Saud bin Abdulaziz University for Health Sciences, Jeddah, SAU; 4 Research Office, King Abdullah International Medical Research Center, Jeddah, SAU; 5 Department of Medicine, Faculty of Medicine, King Saud bin Abdulaziz University for Health Sciences, Jeddah, SAU; 6 Department of Pathology and Laboratory Medicine, Faculty of Medicine, King Abdulaziz Medical City, Ministry of National Guard Health Affairs, Jeddah, SAU; 7 Department of Neurosurgery, King Abdulaziz Medical City, Ministry of National Guard Health Affairs, Jeddah, SAU

**Keywords:** case report, glioblastoma multiforme, intraventricular gbm, intraventricular tumor, pediatric gbm

## Abstract

Intraventricular glioblastoma multiforme (GBM) is an extremely rare disease, with few cases reported in the literature. Here, we present a surgically managed case of an intraventricular GBM in a 54-day-old infant with a long-term follow-up period. The 54-day-old full-term male infant presented to the emergency department due to an increase in head size since the age of 21 days, associated with vomiting after feeding. His past medical history and systemic inquiries were unremarkable. On examination, his head circumference was above the 97th centile. His neurological examination was normal except for hyperreflexia in the lower limbs. Brain imaging showed a supratentorial extra-axial mass occupying the body of the right lateral ventricle. The infant underwent a temporoparietal craniotomy for excision of the intraventricular tumor. Histopathological examination confirmed the diagnosis of pediatric-type glioblastoma, isocitrate dehydrogenase-wild type. The 10-year postoperative follow-up revealed global developmental delay and seizures, which were controlled with levetiracetam. During this period, there was no evidence of tumor recurrence. Intraventricular GBM is considered rare, particularly in the pediatric age group. A high index of suspicion is required for diagnosis. Histopathological examination is necessary to establish the diagnosis and predict the outcome. Despite the poor prognosis associated with intraventricular GBM, our patient demonstrated long-term survival and remained free of recurrence throughout the 10-year follow-up period after surgical excision.

## Introduction

Glioblastoma multiforme (GBM) is a tumor that originates from astrocytes and accounts for 50%-60% of astrocytic tumors. GBM is the most common malignant primary brain tumor, accounting for 12%-15% of intracranial tumors. It is an intra-axial mass, usually localized in the frontotemporal region [1]. Intraventricular tumors are rare and account for about 10% of central nervous system (CNS) neoplasms, with only 13% of these tumors being malignant [2]. Moreover, GBM is considered an extremely rare malignancy in the intraventricular space, typically found in the body of the lateral ventricle or the frontal horn [3,4]. We present the case of a 54-day-old infant with an intraventricular GBM that was surgically resected, with a 10-year follow-up period. This case represents the youngest reported neonate with the longest survival duration.

## Case presentation

**Figure 1:**
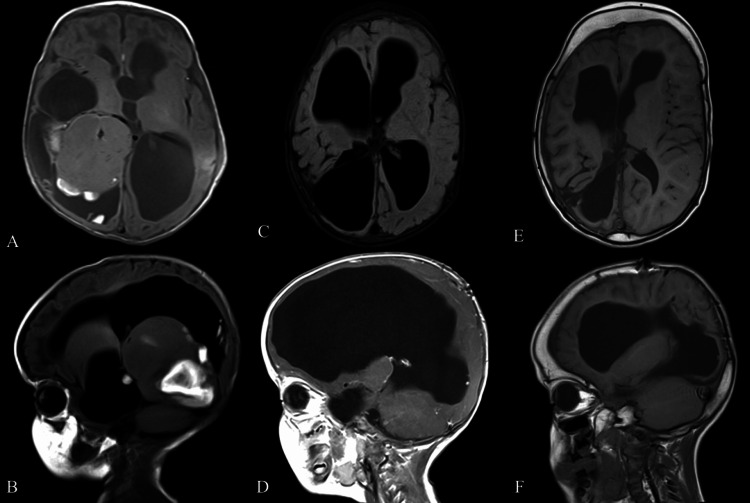
T1 with contrast sequence MRI of the brain: axial (A) and sagittal (B) views showing supratentorial, intraventricular mass (5.2 × 4.4 cm) on the right side, occupying the body of the right lateral ventricle. The mass is heterogeneous in density, including solid and cystic components with lateral, third, and fourth ventricle dilation. Postoperative T1 with contrast sequence MRI of the brain: axial (C) and sagittal (D) views showing extensively enlarged lateral ventricles post-resection of right ventricular GBM. Follow-up: axial (E) and sagittal (F) views of  T1 with contrast sequence MRI of the patient after 5 years showing a reduction in the size of the ventricular system with no evidence of recurrence.

A 54-day-old full-term male infant presented to the emergency department due to an increase in head size since the age of 21 days. Prenatal and postnatal assessments were normal; he was delivered by cesarean section due to the mother's history of previous cesarean deliveries. The increase in head circumference was associated with vomiting after feeding, and the parents denied any abnormal movements, convulsion, apnea, or cyanosis. On examination, the patient’s head circumference was above the 97th centile, with a normal neurological exam except for hyperreflexia in the lower limbs. Brain computed tomography (CT) and magnetic reasoning imaging (MRI) were conducted and showed a supratentorial intraventricular mass (5.2 × 4.4 cm) on the right side, occupying the body of the right lateral ventricle (Figure 1).

**Figure 2:**
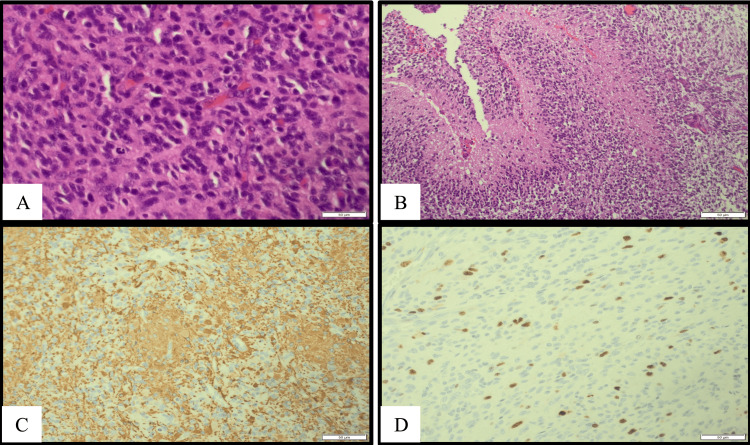
(A and B, 50 μm) H&E-stained slides: infiltrative, cellular, tumor cells with nuclear atypia and brisk mitotic activity. Prominent microvascular proliferation and palisading necrosis (irregular zones of necrosis surrounded by dense accumulations of tumor cells). (C, 50 μm) Delicate processes are evident in GFAP immunostaining. (D, 50 μm) The Ki-67 labeling index is high.

The patient underwent excision of the intraventricular tumor via a temporoparietal craniotomy, with no chemotherapy nor radiotherapy administered. Histopathological examination confirmed a diagnosis of pediatric-type glioblastoma, isocitrate dehydrogenase (IDH)-wild type (WHO grade 4), with no deletion of 1p/19q (Figure 2). Postoperative follow-up of 10 years showed global developmental delay and seizures, which were controlled with levetiracetam. During this period, the patient showed no evidence of recurrence. 

## Discussion

GBM is considered the most common malignant primary brain tumor. It can arise anywhere within the CNS. However, because glial cells are predominantly located in the cerebral hemispheres (86%), with only a few (2.2%) present in the ventricles, GBM most commonly occurs in the frontotemporal area (63%) [2,5]. In contrast, intraventricular GBM is relatively rare.

Based on the latest modification, the onset of symptoms, including, but not limited to, progressive macrocephaly, within the first six weeks of life is now considered indicative of a congenital brain tumor (CBT) [6]. CBTs can also be detected incidentally during routine fetal scans as an intracranial mass, with or without hydrocephalus. They are considered an extremely rare condition, representing about 0.5%-1.9% of all CNS neoplasms in the pediatric population [4]. Although the exact incidence of intraventricular GBM among all CNS tumors remains uncertain, a review of the literature suggests that such cases are rare [7]. The classification of masses affecting the ventricular system is based on their site of origin. Primary tumors originate from the ventricular wall or structures within the ventricle, while secondary tumors originate from adjacent brain parenchyma and grow exophytically into the ventricle. In the past, GBM was diagnosed based on histological features, such as glomeruloid neovascular proliferation and pseudopalisading necrosis. However, the 2021 World Health Organization (WHO) classification of tumors of the CNS now requires immunohistochemistry to confirm the absence of IDH 1 and 2 mutations (IDH-wild type), as well as the absence of mutations in histone 3 (H3-wild type). These criteria have led to the replacement of the term “glioblastoma” with “adult-type diffuse glioma.” For pediatric patients, the term “glioblastoma” is no longer recommended. Instead, this condition is now classified as “pediatric-type diffuse high-grade gliomas (WHO grade 4),” which includes H3-wild type and IDH-wild type [8].

Although GBM is the most common primary malignant CNS tumor in adults, it accounts for only about 10%-15% of cases in the pediatric population [9]. A few cases of intraventricular GBM have been reported in the pediatric age group (Table 1) [7,10-16]. Unlike adult GBM, pediatric GBM is associated with a better prognosis; however, intraventricular GBM is considered to have the poorest prognosis among all CNS tumors and compared to other tumor locations [16]. 

**Table 1:** Review of literature on pediatric intraventricular glioblastoma.

Author	Year	Age in years/gender	Presenting symptoms	Investigation results	Management	Outcome
Wilson and Gardner [16]	1964	5/male	Persistent vomiting and lethargy	Pneumoencephalogram showed that air did not fill the ventricles. Ventriculography showed a large mass within the dilated left trigone	Left parieto-occipital craniotomy and a biopsy specimen was taken	The patient did not recover consciousness. One month later, he died of pneumonia
Guibaud et al. [12]	1997	Male infant	Discovered during antenatal screening	Sonogram revealed an enlarged head with a biparietal diameter exceeding the 95th percentile. Brain MRI of the fetus showed a mass measuring up to 7 cm involving most of the left lateral ventricle	None	According to the parents' decision, no resuscitation maneuvers were attempted, and the infant died 24 hours after birth
Klein and Marchal [13]	2007	9/male	Signs of increased intracranial pressure	Neuroradiological imaging demonstrated a lesion located in the right lateral ventricle	Complete surgical removal of this intraventricular tumor with adjuvant radiotherapy and chemotherapy	A small, local, and asymptomatic recurrence was observed three months later. The child died one year after diagnosis from tumor progression
Baallal et al. [7]	2016	13/male	Headache, vomiting, and left-sided hemiparesis	Brain MRI showed an intraventricular rim-enhancing, heterogeneous mass in the third ventricle infiltrative pattern and around the medial occipital ventricular wall, spreading into the splenium of the corpus callosum and septum pellucidum with obstructive hydrocephalus	Stereotactic biopsy from the mass lesion and insertion of a ventriculoperitoneal shunt	Symptoms of obstructive hydrocephalus were progressively improved. His family refused concomitant chemoradiotherapy for the reason of economic status
Sarsilmaz et al. [14]	2010	16/male	Signs of increased intracranial pressure	Brain MRI showed the tumor-filled posterior body and the occipital horn of the left lateral ventricle, invaded the surrounding parenchyma	Partial resection with adjuvant radiotherapy and chemotherapy	24 months of disease-free survival; however, he experienced a “butterfly” recurrence afterward
Nsir et al. [11]	2016	10/male	Signs of increased intracranial pressure and hemiparesis	Neuroradiological imaging demonstrated a lesion located in the trigone of the lateral ventricle and parietal lobe	Subtotal resection with adjuvant radiotherapy	Died one year later
Tan and Mankad [15]	2018	16/male	Functional decline	Brain MRI showed a large, solid mass within the left lateral ventricle, extending into the left foramen of Monro, resulting in upstream hydrocephalus	Completely resection with adjuvant chemotherapy and radiotherapy	Remained well at two-year follow-up
Belfquih and Akhaddar [10]	2020	8/female	Headache, blurred vision, and confusion	Brain MRI showed a third ventricular mass lesion with obstructive hydrocephalus	Neuroendoscopic biopsy with adjuvant chemotherapy and radiotherapy	Remained well at six-month follow-up

As ventricular GBM grows slowly within the ventricular system, it can eventually lead to hydrocephalus or cause compression symptoms as it expands into eloquent structures [6]. The most commonly reported symptoms include headaches, increased intracranial pressures, and visual defects. Patients may also experience focal neurological deficits, nausea, disorientation, memory loss, and ataxia, to a lesser extent, depending on which nearby eloquent structures are compressed [11]. MRI is the diagnostic method of choice for determining the origin and extent of the lesion, which aids in the management [14]. Common MRI findings include irregular borders and heterogeneous or ring-like contrast enhancement. A biopsy should be obtained for histopathological examination to establish the diagnosis. The management of intraventricular GBMs depends on the size and location of the tumor. The goal of surgery is to alleviate symptoms and improve survival. Gross total resection and debulking are crucial factors that influence the prognosis of GBM patients [7]. Minimal resection or biopsy is associated with a poor prognosis and a high risk of recurrence, while gross debulking and resection followed by radiotherapy with adjuvant chemotherapy have been shown to improve the quality of life of GBM patients [15].

This study has some limitations, primarily because the included literature evidence comes from case reports which may affect the quality of the study. In addition, immunohistochemical analyses were not available at our institution; therefore, they were not performed or included in this study. This limitation may impact the diagnosis, as other diagnoses, such as diffuse pediatric-type high-grade glioma, cannot be fully excluded as potential differential diagnoses. However, given the rarity of intraventricular GBM in the pediatric population, this study provides the best and latest evidence in the literature. Further studies are needed to better understand the underlying etiologies and treatment modalities for intraventricular GBM in pediatric patients. 

## Conclusions

Intraventricular glioblastoma is considered rare, especially in the pediatric age group. A high index of suspicion is required for accurate diagnosis. Histopathological examination is necessary to establish the diagnosis and predict the outcome.

Although previous literature suggests that intraventricular GBM tumors are associated with a poor prognosis, our patient demonstrated long-term survival and remained free of recurrence throughout the 10-year follow-up period after surgical excision. These findings can help understand the pathological patterns and management considerations in such patient groups.
